# Diarrhea due to SARS-CoV-2-Related Exocrine Pancreatic Insufficiency

**DOI:** 10.1155/2021/9920981

**Published:** 2021-05-21

**Authors:** Harish K. Patel, Jasbir Makker, Ahemd Alemam, Sridhar Chilimuri

**Affiliations:** ^1^Division of Gastroenterology, BronxCare Hospital Center a Clinical Affiliate of Mt Sinai Health Systems and Academic Affiliate of Icahn School of Medicine, Bronx, NY 10457, USA; ^2^Department of Medicine, BronxCare Hospital Center a Clinical Affiliate of Mt Sinai Health Systems and Academic Affiliate of Icahn School of Medicine, Bronx, NY 10457, USA

## Abstract

Gastrointestinal symptoms, especially diarrhea, are common with novel coronavirus SARS-CoV-2 infection. Angiotensin-converting enzyme-2 (ACE-2) receptors are heavily expressed in enterocytes and serve as entry receptors for SARS-CoV-2. ACE-2 receptors may also be responsible for pancreatic injury in patients infected with SARS-CoV-2. Diarrhea associated with SARS-CoV-2 is usually believed to be due to viral invasion of enterocytes. However, exocrine pancreatic insufficiency resulting from SARS-CoV-2 is another plausible mechanism leading to diarrhea in such patients. We present a case series of three SARS-CoV-2-infected patients with predominant respiratory symptoms at presentation who developed diarrhea, and further fecal analysis revealed exocrine pancreatic insufficiency as the underlying mechanism.

## 1. Introduction

The novel coronavirus (SARS-CoV-2) was first identified in Hubei Province, China. The World Health Organization (WHO) has declared the SARS-CoV-2 outbreak as a pandemic with more than 5 million affected across the globe. SARS-CoV-2 binds to the angiotensin-converting enzyme-2 (ACE-2) receptor to enter host cells [1]. The respiratory tract involvement leading to pneumonia and acute respiratory distress syndrome (ARDS) is the leading cause of mortality in SARS-CoV-2 infection. Patients with the hypertension, diabetes mellitus, and old age are expected to have worse outcomes [2].

Gastrointestinal symptoms besides respiratory manifestations are common in patients with SARS-CoV-2 infection. The proportion of patients with gastrointestinal manifestations varies in different studies; however, it is estimated that around 35% to 40% of patients with SARS-CoV-2 may be affected [3]. Nausea, vomiting, and diarrhea are the common gastrointestinal symptoms. The incidence and prevalence of SARS-CoV-2-related diarrhea is well reported. However, the mechanisms of diarrhea remain unclear. Infected individuals may have prolonged viral shedding. A recent meta-analysis has revealed that after initial infection, around 48% of patients continue to have persistent shedding of SARS-CoV-2 virus particles in their feces.

We present three cases of SARS-CoV-2 pneumonia managed at our institution. All three patients also had diarrhea, with one developing diarrhea after discharge. The presence of fecal fat, low fecal elastase, and signs of pancreatic injury led us to manage this group of patients with pancreatic enzyme replacement. Resolution of diarrhea with pancreatic enzyme supplements suggests association of SARS-CoV-2-induced diarrhea and exocrine pancreatic insufficiency.

## 2. Case Presentation

### 2.1. Case 1

A 38-year-old man with no major comorbid conditions presented to the emergency room (ER) with the symptoms of cough of 3 days' duration. He had nausea and loss of appetite.

He also reported subjective fevers at home. Upon presentation, he denied shortness of breath, but worsening cough prompted him to seek medical attention.

On initial evaluation, he did not appear dyspneic and his oxygen saturation was 92% on room air. His initial chest roentgenogram (X-ray) revealed the bilateral interstitial infiltrates. He was hospitalized for monitoring of his respiratory status. His nasopharyngeal PCR swab for SARS-CoV-2 was positive, and he was given oral hydroxychloroquine and azithromycin. His laboratory parameters were significant for aspartate aminotransferase (AST) of 44 U/dL, lactate dehydrogenase (LDH) of 410 U/dL, C-reactive protein (CRP) of 32 mg/dL, and ferritin of 900 ng/dL. His random blood glucose and fasting glucose levels were 176 gm/dl and 116 gm/dl, respectively. His hemoglobin A1C (HbA1C), performed 2 weeks after presentation, was 6.1%, suggesting previously undiagnosed prediabetes. He remained clinically stable and had no worsening of his respiratory status, his oxygen saturation on room air improved to 96%, and he was discharged from the hospital on day 6.

A week after the hospital discharge, he reported new-onset diarrhea and mild abdominal pain. His symptoms started 2 days after discharge. He stated that his abdominal pain radiated to back and improved on bending forward. There was no worsening of abdominal pain with meals. His stool consistency was loose and he had 5 to 6 bowel movements per day. He denied any blood in the stool. He also denied nausea and vomiting. Stool *Clostridium difficile* toxin and glutamate dehydrogenase antigen (GDH) were negative. Fecal leucocyte stain was negative, but fecal fat was reported to be positive. Repeat evaluation of fecal fat remained positive. Due to persistent abdominal pain, serum lipase was performed, and it was found to be approximately 3 times the upper limit of normal (176 U/dL). He declined computed tomography (CT) of the abdomen. He had an ultrasound of his abdomen performed previously 6 months earlier which revealed fatty liver. Fecal elastase test was reported to be 110 *μ*/gm of stool. Given the possibility of exocrine pancreatic insufficiency (EPI), he was initiated on pancreatic enzyme replacement with 2 tablets of delayed release pancrelipase capsule comprising 36000 (36 K) units lipase with each meal. Oral omeprazole 40 mg once a day was also added. His diarrhea resolved with the initiation of pancreatic enzyme supplementation. He reported recurrence of diarrhea with discontinuation of pancreatic enzyme supplementation. Symptoms once again resolved with resumption of pancreatic enzyme therapy.

### 2.2. Case 2

A 53-year-old man with a history of hypertension and obesity presented with cough, fever, and shortness of breath of 4 days' duration. He also reported diarrhea for 2 days. He had 5-6 episodes of nonbloody watery bowel movements daily. The worsening shortness of breath prompted him to seek medical attention. He drank alcohol occasionally and had reported 2 to 4 drinks in one year. He was a nonsmoker. On initial assessment, he had mild respiratory distress, with oxygen saturation of 88% on room air, pulse of 112 beats per minute, and blood pressure of 164/92 mmHg. His chest X-ray revealed perihilar infiltrates. Electrocardiogram showed left ventricular hypertrophy, and subsequent echocardiogram revealed ejection fraction of 62%.

He was hospitalized for monitoring of his respiratory status. He was initiated on oral hydroxychloroquine and azithromycin. He required venti-mask with the FiO_2_ of 50% to achieve oxygen saturation of 96%. His laboratory results at the time of presentation showed lactate dehydrogenase (LDH) of 450 U/dL, CRP of 42 mg/dl, ferritin of 870 ng/dL, and d-dimer of 380 U/dL. His alanine aminotransferase (ALT) and aspartate transaminase (AST) levels were 88 U/dL and 65 U/dL, respectively. He had random blood glucose of 98 gm/dL at the time of presentation. Fecal leucocytes were negative. Stool tested negative for the GDH and *Clostridium* toxin A and B. However, his fecal fat was positive. His serum lipase was elevated to 132 mg/dL on 5th day of hospitalization. He did not report any abdominal pain, but CT scan of the abdomen with IV contrast done for the evaluation of the elevated lipase revealed findings suggestive of mild acute pancreatitis. There was no pancreatic necrosis or peripancreatic collection. He was also noted to have liver steatosis with a fat sparing liver lesion. The ultrasound of the abdomen did not reveal any gallbladder stones. His pneumonia improved, and he was discharged home. He reported persistent diarrhea. He was initiated on pancreatic enzyme replacements. The result for fecal elastase could not be obtained due to insufficient stool specimen collection. Patient reported resolution of diarrhea after initiation of oral pancreatic replacement therapy. He noticed recurrence of diarrhea with discontinuation of the pancreatic enzyme replacement therapy.

### 2.3. Case 3

A 69-year-old man with a history of hypertension and compensated diastolic congestive heart failure (CHF) presented with cough, shortness of breath, malaise, fever, and severe myalgia of 5 days' duration. He was a nonsmoker and denied any alcohol use in the past. He had oxygen saturation of 78% on room air, and his X-ray chest revealed bilateral infiltrates. His nasopharyngeal swab test was positive for SARS-CoV-2. He was monitored in the critical care setting and required high-flow oxygen. His lactate dehydrogenase (LDH) level was 680 U/dL, ferritin was 972 ng/dL, and CRP was 56 mg/dl, and his liver function test revealed elevated transaminases with ALT and AST of 68 U/dL and 92 U/dL, respectively. He also developed acute renal insufficiency with a serum creatinine of 3.1 mg/dL. His random blood glucose was 210 gm/dL and fasting blood glucose was 172 mg/dL. He was given amoxicillin/clavulanic acid, azithromycin, and hydroxychloroquine. Four days into his hospitalization, he developed periumbilical abdominal pain and nonbloody water diarrhea with 5 to 6 bowel movements per day. Initially, diarrhea was considered to be a SARS-CoV-2-related symptom. Stool test for *Clostridium difficile* toxin was negative. His fecal leucocyte was negative as well; however, the fecal fat stain test was positive. His serum lipase level was elevated (189 mg/dl). His respiratory status precluded from obtaining a CT scan of the abdomen. He was empirically managed with pancreatic enzyme replacement for possible exocrine pancreatic insufficiency. He was given oral pancreatic enzyme supplement 4 capsules with each of them consisting of 8000 units of lipase. His symptoms improved after one day for the pancreatic enzyme replacement initiation. His respiratory status improved, and he was discharged with a follow-up tele-health appointment. Patient was lost to follow-up upon discharge.

## 3. Key Features of the Presented Cases

The presented patients in our case series had positive nasopharyngeal swabs for the SARS-CoV-2 virus.  Table 1 highlights the key comparative features. Respiratory symptoms were predominant at presentation. The cutoff values of LDH and CRP to gauge the severity of COVID-19 at presentation vary in the literature [4]. Patients with LDH above 250 mg/dl and CRP above 11 mg/dl can be considered to have moderate to severe presentation. Accordingly, our cases would be deemed to have moderate to severe presentation. The onset of diarrhea was not uniform in all cases; however, all patients had positive fecal fat study. There was no significant risk factor for chronic pancreatitis in any of these patients. Serum lipase was noted to be elevated almost twice the upper limit of the normal, and only one patient (Case 2) among these three patients had the imaging study performed to evaluate the pancreas.

## 4. Discussion

Diarrheal illness in a patient with SARS-CoV-2 can be multifactorial. Diarrhea can be differentiated into three categories based on its pathophysiology: secretory, malabsorptive, and inflammatory [5]. Performing fecal fat and fecal leucocyte tests upon initial evaluation helps in categorizing diarrhea [6]. Bacterial infections that cause inflammatory and exudative damage lead to detection of leucocytes in stool [7]. Most viruses and some bacteria, like *Vibrio cholera,* are secretory in nature, and hence, fecal leucocyte test is usually negative. Fat malabsorption may develop due to mucosal infiltrative process, like gastrointestinal amyloidosis or pancreatic insufficiency.

SARS-CoV-2 has been isolated from stool specimen of affected patients presenting with diarrheal illness [8]. All three patients in our case series had established diagnosis of SARS-CoV-2 infection through nasopharyngeal swab testing, but routine stool RT-PCR was not available. Prolonged fecal viral shedding in the infected patients has been reported [9]. Current literature has focused on proportion of patients with gastrointestinal involvement and duration of viral SARS-CoV-2 fecal shedding [10]. However, this does not explain diarrheal symptoms. ACE-2 receptors expressed in the gastrointestinal tract appear to be have an important role in SARS-CoV-2-related diarrheal illness [11]. Endoscopic biopsies reveal the presence of inclusion body and the ACE-2 expression in the stomach, duodenum, and small bowel [12]. These findings may explain the cause of diarrhea in some patients.

Other studies showed that RNA messenger levels for the ACE-2 is higher in pancreas than in lungs [13]. SARS-CoV-2 infection can cause pancreatic damage through the ACE-2 receptors expressed in both exocrine glands and islets of the pancreas [13], and worsening of diabetes has been reported after COVID-19. ACE-2 is also expressed in parathyroid, pituitary, thyroid, and adrenal glands [14]. It is plausible that some patients may develop exocrine deficiency as seen in our patients who had resolution of symptoms with pancreatic enzyme replacements.

However, fecal fat was positive in all the three patients. In 2009, Yang et al. reported acute diabetes mellitus due to SARS-CoV-2 binding to the islet cell receptors [15]. Reports indicate that 17% of patients with SARS-CoV-2 infection can have pancreatic injury, as diagnosed by the serum lipase evaluation [16]. There have been no reports of SARS-CoV-2-related exocrine pancreatic insufficiency, but it remains plausible. The elevated levels of the serum lipase suggest pancreatic injury in our three patients. Acute pancreatitis on imaging study of one of these patients further confirmed the diagnosis.

Quantitative fecal fat studies are required to arrive a diagnosis of steatorrhea [17]. Fat ingestion affects the fecal fat analysis, and dietary fat intake should be taken into account while interpreting fecal fat results [18]. Unfortunately, in our case series, we did not have quantitative fecal fat or the dietary fat intake analysis. Fecal elastase evaluates pancreatic acinar axis and has a very high specificity for evaluating the pancreatic exocrine insufficiency [19]. Patients with fecal elastase of 100 *μ*/g to 200 *μ*/g are considered to have mild to moderate pancreatic insufficiency. Fecal elastase is a reliable and noninvasive parameter to assess pancreatic exocrine function [20]. Based on the presence of fecal fat and fecal elastase positive result, we considered exocrine pancreatic deficiency a possible etiology of diarrhea.

Steatorrhea has several etiologies and is broadly classified into (i) exocrine pancreatic insufficiency, (ii) bile acid deficiency, and (iii) small bowel disease [21]. Small bowel infiltration with SARS-CoV-2 virus can possibly explain fecal fat; however, the absence of fecal leucocytes makes it less likely. The diagnosis of the exocrine pancreatic insufficiency is often clinical and is supported by response to pancreatic enzyme supplementation [22]. Our three patients developed symptoms after SARS-CoV-2 infection with evidence of pancreatic insufficiency. There was complete resolution of symptoms with pancreatic enzyme supplements strengthening the diagnosis pancreatic exocrine deficiency. Symptoms even recurred upon discontinuation of enzyme supplement therapy.

Many patients with COVID-19 receive antibiotics, and hence, antimicrobial-induced diarrheal illness and *Clostridium difficile* infection must be excluded. The fecal leucocyte and toxin for *Clostridium difficile* were negative in our patients.

## 5. Conclusion

Acute diarrheal illness and abdominal pain are common in patients with SARS-CoV-2 infection. Although uncommon, exocrine pancreatic insufficiency should be considered in the differential diagnosis of diarrhea in patients with SARS-CoV-2. Further studies are needed to understand the nature of pancreatic injury in COVID-19 patients.

## Figures and Tables

**Table 1:** Comparison of key presenting features.

	Case 1	Case 2	Case 3
Demographic	A 38-year-old man with no significant medical condition	A 53-year-old man with prior history of hypertension and obesity	A 69-year-old man with hypertension and diastolic CHF
Social history	Smoker and alcohol use of around 4 to 6 drinks in a week for 10 years	Nonsmoker and alcohol use of 2–4 drinks in one year	No smoking and no alcohol use
COVID-19 presentation	Respiratory symptoms of cough and no shortness of breath	Cough, shortness of breath, and fever	Cough, shortness of breath, malaise, fever, and severe myalgia; he required high-flow oxygen during hospitalization
Diarrheal symptoms	Developed 2 to 3 days after hospital discharge	Present at the time of presentation	Developed diarrhea during hospitalization
Stool studies	Fecal fat present; fecal leucocyte negative	Fecal fat present; fecal leucocyte negative	Fecal fat present; fecal leucocyte negative
Fecal elastase (*μ*/dl)	110	Insufficient specimen	Could not be performed
Lactate dehydrogenase (U/dL)	410	450	680
C-reactive protein (mg/dL)	32	42	56
Pancreatic endocrine evaluation	Prediabetic	Random glucose of 98 gm/dL	Prediabetic
Time to respond to pancreatic enzyme supplements	1 day	1 day	1 day
Recurrence of symptoms with stopping pancreatic enzyme supplements	Present	Present	No interruption of therapy
